# MicroRNA miR-145-5p regulates cell proliferation and cell migration in colon cancer by inhibiting chemokine (C-X-C motif) ligand 1 and integrin α2

**DOI:** 10.1080/21655979.2021.2000243

**Published:** 2021-12-03

**Authors:** Wei Zhuang, Tao Niu, Zhen Li

**Affiliations:** Department of Gastroenterology, People’s Hospital of Dongxihu District, Wuhan, Hubei, China

**Keywords:** miR-145-5p, CXCL1, ITGA2, colon cancer, proliferation, migration

## Abstract

Colon cancer (CC), which has high morbidity and mortality, can be regulated by microRNAs. This study aimed to investigate the regulatory function of microRNA miR-145-5p in CC cells. Bioinformatics analysis was used to screen key genes in CC. The expression of miR-145-5p, chemokine (C-X-C motif) ligand 1 (CXCL1), and integrin α2 (ITGA2) in CC was confirmed by quantitative reverse transcription polymerase chain reaction and western blotting. After cell transfection, changes in proliferation and migration in CC cells were detected using the cell counting kit-8 (CCK-8), colony formation assay, and wound healing assay. A luciferase assay was conducted to confirm the interactome of miR-145-5p, CXCL1, and ITGA2 in CC cells. Bioinformatics analysis confirmed that CXCL1 and ITGA2 were key genes in CC. After performing several cell functional experiments, the results confirmed that upregulation of miR-145-5p attenuated proliferation and migration of CC cells. Luciferase assay and western blotting confirmed that CXCL1 and ITGA2 were targets of miR-145-5p, and their expression in CC could be suppressed by miR-145-5p. In conclusion, miR-145-5p is a tumor suppressor in CC and can inhibit the expression of CXCL1 and ITGA2.

## Introduction

Colon cancer (CC), with high morbidity and mortality rates, is widely distributed worldwide [1,2]. In China, people with CC tend to be younger due to changes in lifestyle and diet [3]. Despite major advances in therapeutic strategies, including chemotherapy, surgical resection, and radiotherapy, it has been suggested that the number of patients with CC will increase by 60% worldwide by 2030 [4]. Therefore, identification of novel molecular markers is necessary for CC therapy.

MicroRNAs (miRNAs) of ~22 bp are common non-coding RNAs that often participate in the progression of human cancers [5]. miRNAs are regulated by competitive endogenous RNA (ceRNA) [6]. The ceRNA mechanism identifies specific miRNA-binding sites on the target genes of miRNAs, thereby regulating the expression of target genes [6]. miR-145-5p, a member of the p53-tumor suppressor network, acts as a post-transcriptional regulator of many cancer-related genes [7]. Previous studies have reported that miR-145-5p is a suppressor in breast cancer by regulating SOX2 [8], gastric cancer by regulating KLF5 [9], and bladder cancer by regulating TAGLN2 [10]. However, the downstream role of miR-145-5p in key target genes has not been fully explored. Here, we investigated the effect of miR-145-5p on CC *in vitro*, which was shown to inhibit angiogenesis in CC by regulating the EGFR-associated signaling pathway [11].

Chemokine (C-X-C motif) ligand 1 (CXCL1), a member of the G protein-coupled receptor family, is overexpressed in colorectal cancer to facilitate metastasis and progression [12,13]. Integrin α2 (ITGA2) is highly expressed in multiple cancers to regulate tumorigenesis [14–16]. In colorectal cancer, ITGA2 was also shown to be an oncogene that was targeted by miR-16-5p [17]. However, the regulatory mechanism upstream of CXCL1 and ITGA2 in CC is still not explored.

According to a previous study and bioinformatics analysis, we suspected that miR-145-5p might regulate CC progression by targeting CXCL1 and ITGA2. Hence, this study aimed to investigate the interaction among miR-145-5p, CXCL1, and ITGA2 in CC progression using cell functional experiments. Our findings may provide potential molecular markers for CC therapy.

## Materials and methods

### Bioinformatics analysis

GEPIA is a database that includes differentially expressed genes in CC samples, and GSE126095 is a microarray that includes the expression of genes and miRNAs in CC samples. Genes upregulated in CC, with adjusted p < 0.05, and logFC > 2 values, were screened using GEPIA and GSE126095. STRING was then applied to GO enrichment. Finally, TargetScan was used to predict the miRNAs targeting the key genes. At the same time, the downregulated miRNAs in CC samples were screened using GSE126095 with adjusted p < 0.05 and logFC < −1.

### Clinical sample collection and cell culture

The use of CC tissues and adjacent normal tissues from 36 patients diagnosed with CC in our hospital between April 2019 and November 2020 was approved by the ethics committee of the People’s Hospital of Dongxihu District. All volunteers signed an informed consent statement, and their clinical characteristics are listed in Supplementary Table 1.

All cell lines used in this study, including CC cell lines (SW620, SW480, and HCT116) and the human normal colon cell line FHC, were obtained from BeNa Culture Collection (China). The SW480 cell line was incubated in DMEM, whereas the other cell lines were incubated in RPMI-1640 medium. All media were supplemented with 10% FBS, and the cells were incubated at 37°C with 5% CO_2_.

### Cell transfection

We transfected 1 × 10^5^/mL SW620 and HCT116 cells with miR-145-5p mimic (miR10000437-1-5, RiboBio, China), mimic-NC (miR1N0000001-1-5, RiboBio,China),miR-145-5pinhibitor(miR20000437-1-5, RiboBio, China), and inhibitor-NC (miR2N0000001-1-5, RiboBio, China) at a final concentration of 50 nM using Lipofectamine 2000 (Thermo Fisher Scientific, Inc., USA). Cell transfection was performed at 37°C with 5% CO_2_ and maintained for 48 h for subsequent experiments.

### Quantitative reverse transcription polymerase chain reaction (qRT-PCR)

RNA extraction was conducted using TRIzol reagent (Invitrogen, USA), and RNA reverse transcription was performed using the PrimScript RT reagent Kit (Takara, Japan). After transcription, qRT-PCR was performed using the Applied Biosystems StepOne System (Applied Biosystems, USA). The 2-^ΔΔCt^ method [18] was used to analyze the expression of miRNAs and mRNAs. The mRNA expression was normalized to that of GAPDH, and miRNA expression was normalized to U6. The primer sequences used are listed in Supplementary Table 2.

### Cell proliferation detection

The change in cell proliferation was evaluated using the cell counting-8 kit (CCK-8) and colony formation assays, as described previously [19,20]. In the CCK8 assay, CC cells in the logarithmic growth phase were seeded in 96-well plates at a density of 5,000 cells/well. After transfecting cells for 0, 24, 48, and 72 h, serum-free medium with 10 μl of CCK-8 solution (Beyotime, China) was added to each well and incubated for 4 h at 5% CO_2_ and 37°C. Finally, the absorbance of each well was measured to evaluate the cell proliferation ability using a microplate reader at 450 nm.

For the colony formation assay, cells were digested with trypsin after transfection, and 1,000 cells were seeded into six-well plates and incubated at 37°C with 5% CO_2_. Cell transfection plasmids were added to the cells every 48 h. After 15 days, colonies were stained using 0.1% crystal violet and photographed using an optical microscope to count the number of colonies.

### Cell migration detection

Cell migration ability was detected using a wound healing assay, according to a previous study [21]. CC cells were seeded in six-well plates and cultured until ~100% confluence. Then, the cells were scratched using a 200 μl sterile pipette tip, and PBS was used to remove the exfoliated cells. Next, the cells were incubated for 24 h in a serum-free medium. The wound at 0 and 24 h was photographed using an optical microscope to determine the cell migration rate.

### Luciferase assay

The wild-type (WT) CXCL1 3ʹUTR and ITGA2 3ʹUTR containing the binding sites for miR-145-5p were synthesized and inserted into the pGL3 vectors (Promega, USA). The mutant (MUT) CXCL1 3ʹUTR and ITGA2 3ʹUTR mutated from WT by site-directed mutagenesis were also inserted into the pGL3 vectors. Then, the WT or MUT CXCL1 vectors and WT or MUT ITGA2 vectors were co-transfected with miR-145-5p mimic or mimic-NC into colon cells. After 48 h of co-transfection, the Dual-Luciferase Reporter Assay Kit (Promega) was used to verify firefly luciferase activity and Renilla luciferase activity [19].

### Western blotting

Western blotting was performed as previously described [21]. Total proteins from colon cells were isolated using RIPA lysis buffer (Sigma, USA), and the concentration of isolated proteins was confirmed using a BCA assay kit (Pierce, USA). Then, 20 μg protein was separated using 15% SDS-PAGE gel for CXCL1 protein and 8% SDS-PAGE gel for ITGA2 protein and transferred to PVDF membranes. Next, the membranes were blocked with 5% nonfat milk, and the membranes were incubated overnight at 4°C with the following primary antibodies: CXCL1 (ab206411, Abcam, USA), ITGA2 (ab133557, Abcam, USA), and GAPDH (ab9485, Abcam, USA). Subsequently, the membranes were incubated with the rabbit secondary antibody (ab205718, Abcam, USA) for 3 h at 22–25°C. After washing, the membranes were incubated with an enhanced chemiluminescence kit (Pierce, USA) and placed in an X-ray film cassette. Finally, the X-ray film was placed over the membranes and exposed to visualize the protein signal. The intensity of the bands was quantified using AlphaEase FC version 6.0.2 (Alpha Innotech, USA).

## Statistical analysis

Bioinformatics data were analyzed using GEO2R. The experimental data in this study are shown as mean ± standard deviation from three independent experiments, and the data were analyzed using GraphPad Prism 7.0 (GraphPad Software, Inc., USA). Student’s t-test was performed to analyze differences between two groups, whereas one-way analysis of variance (ANOVA) was conducted to analyze differences among multiple groups. Statistical significance was set at p < 0.05.

## Results

Using bioinformatics analysis, we suspected that miR-145-5p might act as a key miRNA that participates in CC progression by targeting CXCL1 and ITGA2. To further explore our hypothesis, a series of cytological experiments were performed. Our results showed that miR-145-5p attenuated cell proliferation and migration in CC cells, and targeted CXCL1 and ITGA2 in CC cells. Our findings enrich the mechanism of CC progression and may provide molecular markers for CC therapy.

### miR-145-5p might target CXCL1 and ITGA2 to play the key role in CC

**Figure 1.**
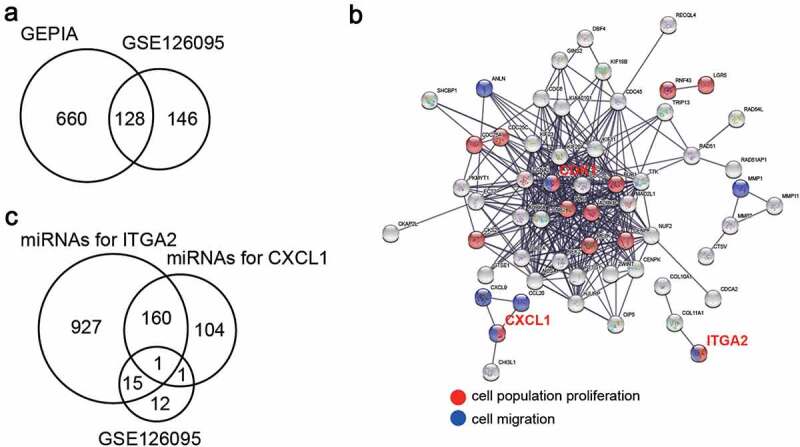
miR-145-5p might target CXCL1 and ITGA2 to regulate cell proliferation and cell migration. (a) 128 upregulated genes overlapped from two databases (GEPIA and GSE126095) with adj.P < 0.05 and logFC>2. (b) GO enrichment for upregulated genes was analyzed by STRING. (c) miR-145-5p was the only miRNA downregulated in colon cancer samples and targeting CXCL1 and ITGA2. TargetScan was used to predict the miRNAs targeting CXCL1 and ITGA2, and the downregulated miRNAs was selected from GSE126095 with adj.P < 0.05 and logFC<-1

A total of 128 upregulated genes in CC samples were screened using GEPIA and GSE126095, which had two mRNA expression profiles with adjusted p < 0.05, and logFC > 2 (Figure 1(a)). After GO enrichment by STRING, it was found that CDK1, CXCL1, and ITGA2 were related to cell proliferation and migration (Figure 1(b)). CXCL1 and ITGA2 caught our attention, since there were many studies on CDK1 in CC [22–24]. TargetScan was then used to predict the miRNAs that could target CXCL1 and ITGA2, and GSE126095, including the miRNA expression profile, was used to screen out the downregulated miRNAs with adjusted p < 0.05, and logFC < −1. As shown in Figure 1(c), only one miRNA (miR-145-5p) overlapped.

### miR-145-5p inhibited proliferation and migration of CC cells

**Figure 2.**
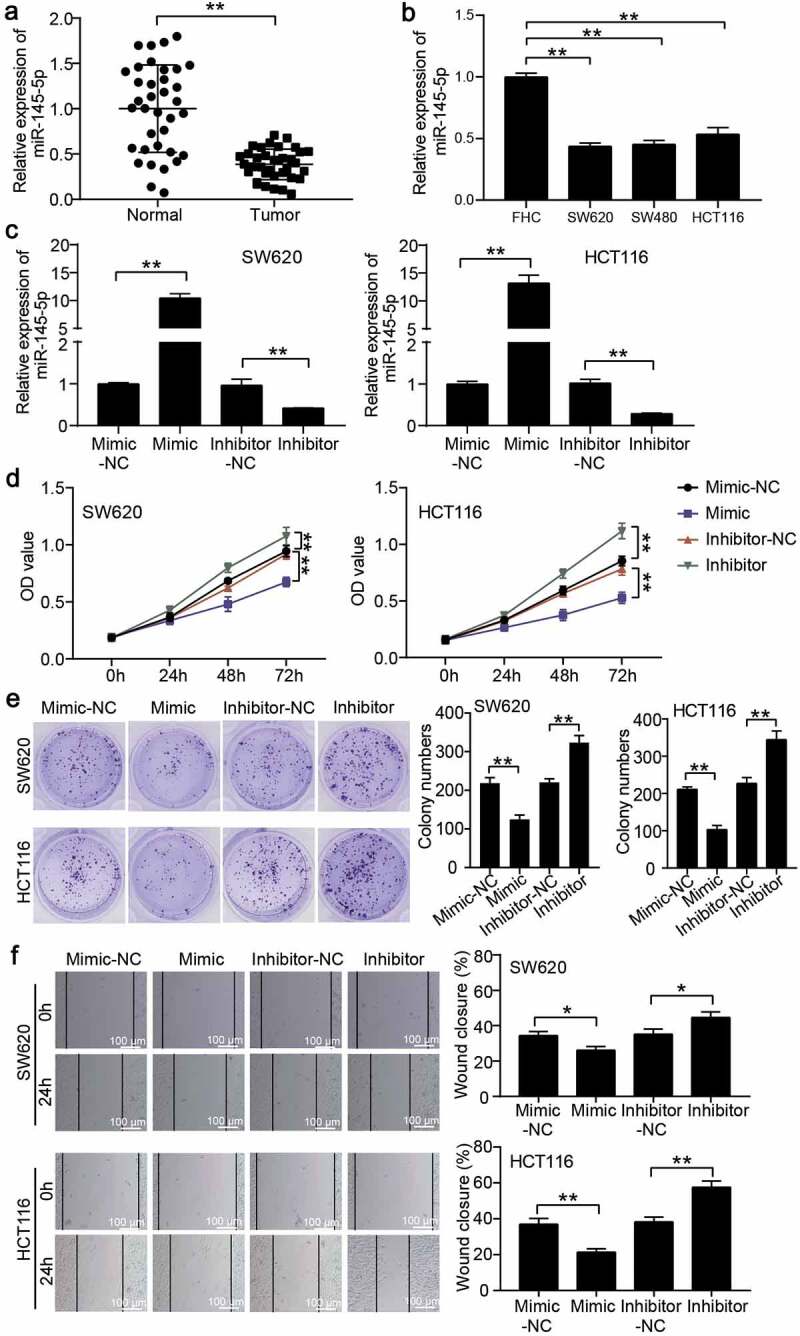
miR-145-5p inhibited proliferation and migration of colon cancer cells. (a) qRT-PCR detected the miR-145-5p expression in adjacent normal tissues and colon cancer tissues. (b) qRT-PCR detected the miR-145-5p expression in human normal colon cell line FHC and colon cancer cell lines (SW620, SW480 and HCT116). (c) The transfection efficiency of miR-145-5p mimic and miR-145-5p inhibitor in SW620 and HCT116 was detected by qRT-PCR. (d-e) The effect of miR-145-5p on cell proliferation was assessed by CCK-8 assay (d) and colony formation assay (e). (f) The effect of miR-145-5p on cell migration was assessed by wound healing assay. NC, negative control. Mimic, miR-145-5p mimic. Inhibitor, miR-145-5p inhibitor. **P < 0.001

To verify the function of miR-145-5p in CC cells, qRT-PCR was performed to confirm miR-148-5p expression in CC tissues and cells. The results showed that miR-148-5p expression was reduced by 60% in CC tissues (Figure 2(a)), and also in CC cells, including SW620, SW480, and HCT116 (Figure 2(b)). Because of similar miR-148-5p expression in SW620 and SW480 cells, we selected SW620 and HCT116 cell lines to assess the function of miR-148-5p. Then, using miR-148-5p mimic and miR-148-5p inhibitor to transfect SW620 and HCT116, the miR-148-5p expression increased by more than 10-fold in the miR-148-5p mimic group and decreased by approximately 70% in the miR-148-5p inhibitor group (Figure 2(c)). Next, CCK8 and colony formation assays were conducted to evaluate changes in cell proliferation, and we found that miR-148-5p overexpression attenuated cell proliferation and miR-148-5p knockdown facilitated cell proliferation (Figures 2(d,e)). The wound healing assay confirmed that miR-148-5p overexpression impaired the migration capability, and miR-148-5p knockdown enhanced the migration capability *in vitro* (figure 2(f)).

### miR-148-5p could target CXCL1 and ITGA2

**Figure 3.**
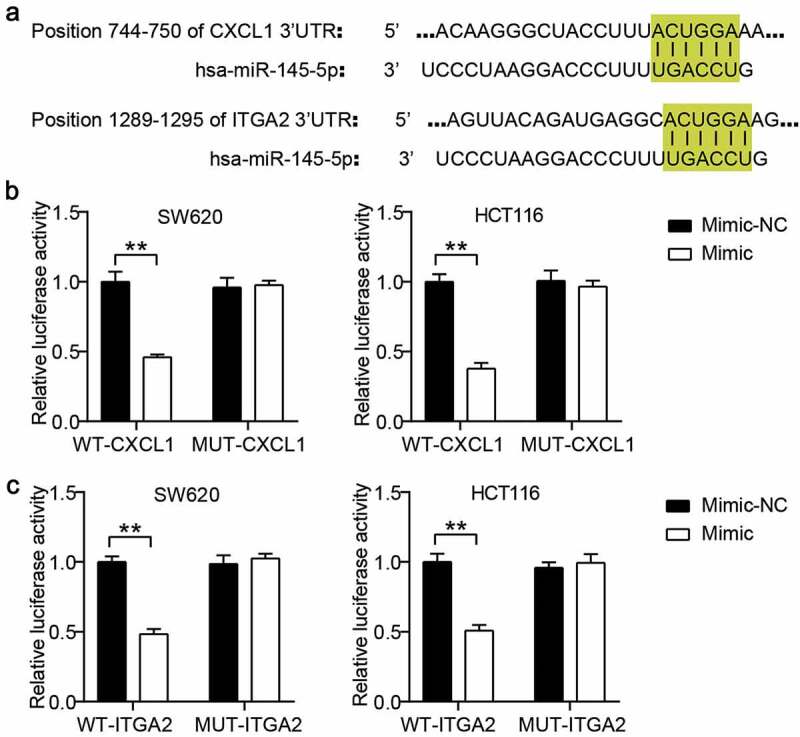
CXCL1 and ITGA2 were the target genes of miR-145-5p in colon cancer cells. (a) The binding sites between CXCL1/ITGA2 and miR-145-5p were predicted by TargetScan. (b) The targeting relationship between CXCL1 and miR-145-5p was proved by luciferase assay. (c) The targeting relationship between ITGA2 and miR-145-5p was proved by luciferase assay. NC, negative control. Mimic, miR-145-5p mimic. **P < 0.001

The binding sites of CXCL1 3ʹUTR and ITGA2 3ʹUTR for miR-145-5p were predicted using TargetScan (Figure 3(a)). Based on the predicted binding sites, we constructed WT CXCL1 or ITGA2 and MUT CXCL1 or ITGA2 vectors to perform a luciferase assay. The results showed that miR-145-5p overexpression reduced the luciferase activities in the WT CXCL1 or ITGA2 groups, but the suppression effect of miR-145-5p mimic on luciferase activity was not observed in the MUT CXCL1 or ITGA2 groups (Figures 3(b,c)).

### miR-148-5p inhibited the expression of CXCL1 and ITGA2

**Figure 4.**
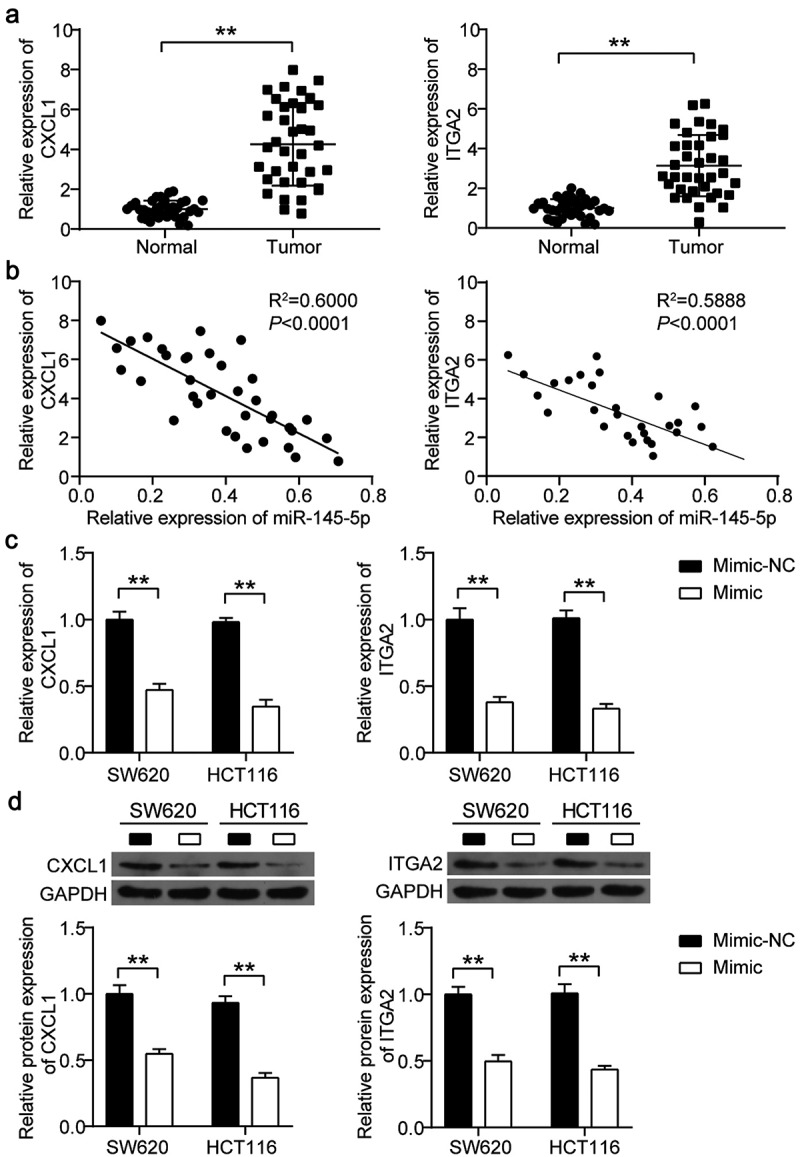
miR-145-5p inhibited the expression of CXCL1 and ITGA2 in colon cancer. (a) The expression of CXCL1 and ITGA2 in adjacent normal tissues and colon cancer tissues was detected by qRT-PCR. (b) The correlation between CXCL1/ITGA2 and miR-145-5p was analyzed by Pearson correlation analysis. (c) The mRNA expression of CXCL1 and ITGA2 in SW620 and HCT116 cells with miR-145-5p mimic was detected by qRT-PCR. (d) The protein expression of CXCL1 and ITGA2 in SW620 and HCT116 cells with miR-145-5p mimic was detected by western blotting. NC, negative control. Mimic, miR-145-5p mimic. **P < 0.001

To further analyze the correlation among miR-148-5p, CXCL1, and ITGA2, we detected the expression of CXCL1 and ITGA2 in CC tissues by qRT-PCR. The results indicated that both CXCL1 and ITGA2 were upregulated by four-fold and 3.5-fold, respectively, in CC tissues (Figure 4(a)). Pearson correlation analysis showed that miR-148-5p expression was negatively correlated with the expression of CXCL1 and ITGA2 in CC tissues (Figure 4(b)). After transfecting the miR-148-5p mimic into SW620 and HCT116 cells, it was found that the expression of CXCL1 and ITGA2 was reduced in the miR-148-5p mimic group (Figure 4(c)). Western blotting also showed that the protein levels of CXCL1 and ITGA2 were reduced by >50% in SW620 and HCT116 cells with miR-148-5p mimic transfection (Figure 4(d)).

## Discussion

CC is an aggressive cancer with high morbidity and mortality rates. Here, we investigated the function of the interactome among miR-145-5p, CXCL1, and ITGA2 in CC. Our study revealed that miR-145-5p expression was reduced in CC, and miR-145-5p inhibited cell proliferation and migration in CC cells. Moreover, we confirmed that CXCL1 and ITGA2 were the target genes of miR-145-5p in CC. Our data indicated that miR-145-5p affected CC progression *in vitro* by inhibiting the two oncogenes CXCL1 and ITGA2.

miRNAs have been reported to inhibit the expression of mRNAs by binding to the 3ʹUTR of target genes, thereby participating in multiple biological processes [25–27]. miR-145-5p, a member of the miRNA family, has been proven to be a tumor suppressor in human cancers such as glioma [28], breast cancer [8], and bladder cancer [10]. In colorectal cancer, Chen et al. identified that miR-145-5p expression was downregulated and miR-145-5p mimic suppressed cell viability, migration, invasion, and epithelial–mesenchymal transition [29]. With respect to CC, only one team from Thuringer et al. [30] explored the function of miR-145-5p in angiogenesis, indicating the inhibitory effect of miR-145-5p on CC. Together with results of the previous study, we further confirmed the role of miR-145-5p in CC using cell functional experiments. Our results were similar to those of previous studies, which showed that miR-145-5p inhibited CC progression by inhibiting proliferation and migration. Furthermore, we demonstrated for the first time that CXCL1 and ITGA2 are the target genes of miR-145-5p in CC cells, suggesting that miR-145-5p affects the malignancy of CC cells by downregulating the levels of CXCL1 and ITGA2.

Because miRNAs play a regulatory role in cancer progression by targeting oncogenes or tumor suppressor genes, we explored the downstream role of miR-145-5p in CC by examining its target genes. Together with bioinformatics analysis and a luciferase assay, CXCL1 and ITGA2 were confirmed as targets of miR-145-5p. Previous studies have shown that CXCL1 is an oncogene in colorectal cancer by enhancing metastatic ability and epithelial-to-mesenchymal transition [12,13,31]. ITGA2 also acts as an oncogene in CC by enhancing proliferation, chemotaxis, and metastasis [32,33]. Here, we explored processes upstream of CXCL1 and ITGA2, and found that miR-145-5p is a common miRNA to regulate the expression of CXCL1 and ITGA2 in CC cells. This was a novel finding.

Our study confirmed the mechanism of action of miR-145-5p in CC by targeting CXCL1 and ITGA2. However, the upstream processes of miR-145-5p involving lncRNAs or circRNAs and the downstream processes of CXCL1 and ITGA2 involving key signaling pathways have not been elucidated and need to be further investigated. In addition, the effect of the interactome among miR-145-5p, CXCL1, and ITGA2 on CC needs to be confirmed *in vivo*.

## Conclusion

In conclusion, overall, our study revealed that miR-145-5p was downregulated in CC, and miR-145-5p inhibited cell proliferation and migration in CC cells. Notably, our study is the first to reveal that miR-145-5p regulates CC progression *in vitro* by targeting CXCL1 and ITGA2. These findings may provide novel insights into the therapy of CC.

## Supplementary Material

Supplemental Material

## Data Availability

The datasets used and/or analyzed during the current study are available from the corresponding author on reasonable request.

## Highlights

- miR-145-5p attenuated the proliferation and migration of colon cancer cells.
- miR-145-5p could target CXCL1 and ITGA2 in colon cancer cells.
- miR-145-5p attenuated malignancy of colon cancer cells by inhibiting CXCL1 & ITGA2
