# Supplementary material for: MicroRNA miR-145-5p regulates cell proliferation and cell migration in colon cancer by inhibiting chemokine (C-X-C motif) ligand 1 and integrin α2
Source: Bioengineered. 2021 Dec 3;12(2):9909–17. doi: 10.1080/21655979.2021.2000243 (PMC8810145; doi:10.1080/21655979.2021.2000243)
Supplement: Supplemental Material [file KBIE_A_2000243_SM8940.zip › supplementary/supplementary table 1.docx]

Supplementary Table1. Clinical characteristics of colon cancer patients in this study.

| Variable | Number (Total=36) | Percentage (%) |
| --- | --- | --- |
| Age (years) |  |  |
| ≥70 | 20 | 55.56 |
| <70 | 16 | 44.44 |
| Gender |  |  |
| Male | 25 | 77.78 |
| Female | 11 | 22.22 |
| Lymph node metastasis |  |  |
| Yes | 21 | 58.33 |
| No | 15 | 41.67 |
| TNM stage |  |  |
| I | 6 | 16.66 |
| II | 15 | 41.67 |
| III | 10 | 27.78 |
| IV | 5 | 13.89 |
