# Supplementary material for: MicroRNA miR-145-5p regulates cell proliferation and cell migration in colon cancer by inhibiting chemokine (C-X-C motif) ligand 1 and integrin α2
Source: Bioengineered. 2021 Dec 3;12(2):9909–17. doi: 10.1080/21655979.2021.2000243 (PMC8810145; doi:10.1080/21655979.2021.2000243)
Supplement: Supplemental Material [file KBIE_A_2000243_SM8940.zip › supplementary/supplementary table 2 (1).docx]

Supplementary Table 2: The sequences of primers used for Quantitative Real-time PCR.

| Gene | Primer sequence |
| --- | --- |
| miR-145-5p | F: 5′-GTCCAGTTTTCCCAGGAATC-3′ |
|  | R: 5′-AGAACAGTATTTCCAGGAAT-3′ |
| CXCL1 | F: 5’-CGCTACAGCGACGTGAAGAA-3’ |
|  | R: 5’-GTTCCAGGCGTTGTACCAC-3’ |
| ITGA2 | F: 5’-CACAACGGGTGTGTGTTCTGAC-3′ |
|  | R:5’-TATTTGATTCATCACACACAACCAC-3′ |
| U6 | F: 5′-CTCGCTTCGGCAGCACA-3′ |
|  | R: 5′-AACGCTTCACGAATTTGCGT-3′ |
| GAPDH | F: 5′-ATGTCGTGGAGTCTACTGGC-3′ |
|  | R: 5′-TGACCTTGCCCACAGCCTTG-3′ |
